# Prognostic value of cardiopulmonary exercise test in patients with acute myocardial infarction after percutaneous coronary intervention

**DOI:** 10.1038/s41598-024-66963-5

**Published:** 2024-07-15

**Authors:** Zhengyan Li, Baochao Fan, Yifan Wu, Haomiao Rui, Yongcun Hu, Yingchun Gu, Juncai Bai, Liming Lu, Dongwei Wang

**Affiliations:** 1 Department of Cardiac Rehabilitation, Zhengzhou Central Hospital Affiliated to Zhengzhou University, Zhengzhou, 450000 China; 2 Clinical Research and Big Data Center, South China Research Center for Acupuncture and Moxibustion, Medical College of Acu-Moxi and Rehabilitation, Guangzhou University of Chinese Medicine, Guangzhou, 510120 China; 3 School of Physical Education and Health, Guangzhou University of Chinese Medicine, Guangzhou, China; 4 Henan Provincial Hospital of Traditional Chinese Medicine, Zhengzhou, China; 5 Anyang District Hospital, Anyang, China

**Keywords:** Acute myocardial infarction, Percutaneous coronary intervention, Cardiopulmonary exercise test, Prediction model, Risk factors, Experimental models of disease, Outcomes research

## Abstract

To determine the independent risk factors of cardiopulmonary exercise test (CPET) parameters related to adverse prognostic events within 5 years in patients undergoing percutaneous coronary intervention (PCI) for acute myocardial infarction (AMI), and establish a prediction model for the occurrence of adverse events within 5 years to provide a reference for cardiac rehabilitation training. From August 2015 to December 2021, patients who underwent PCI for AMI and completed CPET within 1–2 weeks after surgery before discharge from the Department of Cardiovascular Medicine of Zhengzhou Central Hospital Affiliated to Zhengzhou University, Henan Provincial Hospital of Traditional Chinese Medicine, and Anyang District Hospital were selected as participants. Univariate and multivariate analyses were used to screen for independent risk factors associated with 5-year adverse events. Feature importance was interpreted using SHapley Additive exPlanations (SHAP), and a logistic regression model was established for prediction. A receiver operating characteristic (ROC) curve was constructed to evaluate the performance of the prediction model. Calibration was assessed by the Hosmer–Lemeshow test and the calibration curve. In total, 375 patients met the inclusion criteria. Based on whether adverse events occurred during the 5-year follow-up period, the patients were divided into two groups: the event group (n = 53) and the non-event group (n = 322). Peak oxygen uptake (peakVO_2_), carbon dioxide ventilation equivalent slope (VE/VCO_2_slop), and peak end-tidal carbon dioxide partial pressure (PETCO_2_) were three independent risk factors for re-acute myocardial infarction (re-AMI), heart failure (HF), and even death after PCI for AMI (*P* < 0.05). The SHAP plots demonstrated that the significant contributors to model performance were related to peakVO_2_, VE/VCO_2_slop, and PETCO_2_. The risk of adverse events was significantly reduced when the peakVO_2_ was ≥ 20 mL/kg/min and the VE/VCO_2_slop was < 33. The ROC curves of the three models were drawn, including the no-event and event groups, re-AMI group, and HF group, which performed well, with AUC of 0.894, 0.760, and 0.883, respectively. The Hosmer–Lemeshow test showed that the three models were a good fit (*P* > 0.05). The calibration curve of the three models was close to the ideal diagonal lines. CPET parameters can predict the prognosis of adverse events within 5 years after PCI in patients with AMI and provide a theoretical basis for cardiac rehabilitation training.

## Introduction

Acute myocardial infarction (AMI) is a serious form of myocardial ischemia and necrosis caused by acute coronary artery stenosis or occlusion. AMI is a major cardiovascular disease that poses a serious threat to human health and is characterized by high prevalence, rapid progression, and high mortality, all of which seriously affect the quality of life of patients and aggravate their medical burden^1,2^. Although percutaneous coronary intervention (PCI) can improve the in-hospital survival rate of patients with myocardial infarction, the incidence of in-stent restenosis remains at 5.0%, and there are a series of cardiac remodeling evolutions that can lead to re-myocardial infarction, heart failure (HF), or even death. Therefore, managing the adverse outcomes after AMI and way of life has become a concern in cardiac rehabilitation.

Previous studies have shown that cardiopulmonary exercise test (CPET) parameters have an extremely good predictive value for the prognosis of patients with cardiovascular disease^3,4^. Nadruz et al.^5^ showed that peak oxygen uptake (peakVO_2_) and the carbon dioxide ventilation equivalent slope (VE/VCO_2_slop) can provide prognostic value for patients with HF; another study has shown that peak end-tidal carbon dioxide partial pressure (PETCO_2_) has a greater prognostic ability than peakVO_2_ for adverse events in patients undergoing cardiac resynchronization therapy (CRT)^6^. Moreover, Nakade et al.^7^ showed that pulse pressure differences in exercise tests can accurately predict cardiovascular death in patients with HF. In addition, the trajectory of oxygen uptake in an exercise test^8^ and the size of the oscillatory ventilation ring^9^ can predict the prognosis of patients with HF. The latest research in China used CPET indicators to predict the prognosis of patients with anxiety after PCI for coronary heart disease^10^. However, to date, no study has used CPET to predict the rate of re-acute myocardial infarction (re-AMI) and the risk of HF and death in patients with AMI after PCI, which need to be further studied.

Therefore, this study aimed to observe and analyze the predictive ability of CPET parameters for the re-AMI rate, incidence of HF after AMI, and death in patients with AMI after PCI, as well as aiming to build a prognostic model of patients with AMI after PCI, providing a reference for the postoperative management and rehabilitation of patients with AMI.

## Methods

### Study design and participants

Patients who underwent PCI for AMI and completed CPET within 1–2 weeks after surgery at the Department of Cardiology, Zhengzhou Central Hospital Affiliated to Zhengzhou University, Henan Province Hospital of Traditional Chinese Medicine, and Anyang District Hospital from July 2015 to December 2021 were analyzed. Modeling data were obtained from Zhengzhou Central Hospital Affiliated to Zhengzhou University, Henan Province Hospital of Traditional Chinese Medicine, and Anyang Regional Hospital. The specific inclusion criteria were as follows: patients who (i) met the diagnostic criteria of the 2018 AMI general definition (fourth edition) guide, (ii) were aged 18–80 years, and (iii) underwent PCI and completed CPET. The exclusion criteria were as follows: (i) ejection fraction < 50.0% and (ii) AMI combined with a malignant tumor, pulmonary hypertension, and other diseases affecting the patient’s life. The study complies with the Declaration of Helsinki.

**Figure 1:**
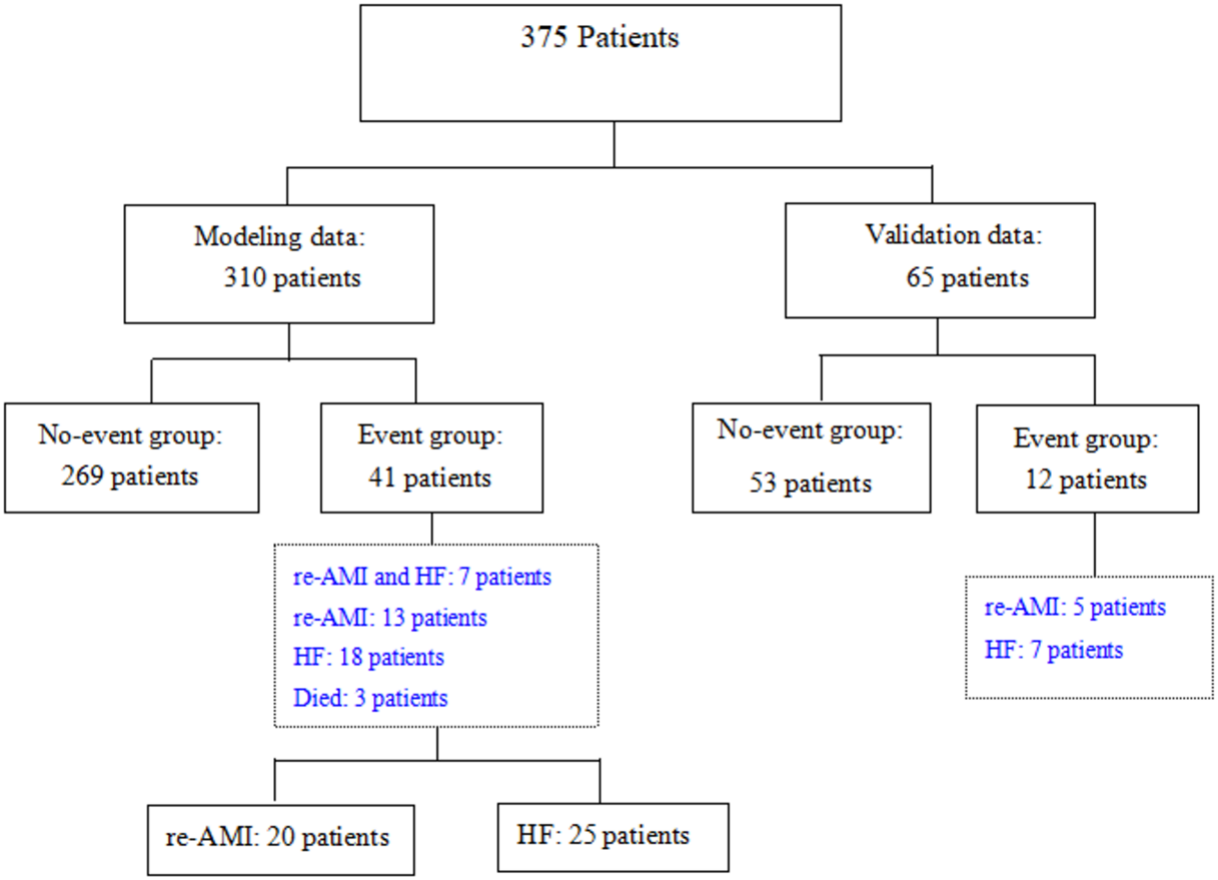
Research group design. *re-AMI* re-acute myocardial infarction, *HF* heart failure.

Overall, this study included 375 patients who met the inclusion criteria, with an average follow-up duration of 5 years. The modeling data were divided into 269 patients in the no-event group and 41 patients in the event group, including seven patients with re-AMI complicated with HF, 13 patients with re-AMI, 18 patients with HF, and three deaths (see Fig. 1), including one due to a car accident and two due to cardiovascular death.

### Ethics approval

This study was approved by the Zhengzhou Central Hospital Affiliated to Zhengzhou University (approval number: 202462). Informed consent was obtained from all the participants for primary data collection. All research data were anonymous or de-identified to protect the participants’ privacy. We did not provide any remuneration to participants who provided complete and valid responses.

### CPET

All participants in this study were informed of the relevant precautions before CPET and signed an informed consent form. The CPET was performed after a professional cardiovascular physician evaluated the patient’s physical condition. Cardiopulmonary function was assessed using the power bicycle ramp protocol. Before the first test, after the tester was restarted, the gas flow rate sensor and gas analyzer were calibrated to measure oxygen and carbon dioxide. The patient was connected to the test system after wearing the device and began to pedal after resting for 3 min. The baseline metabolic and static cardiopulmonary function data were collected. Subsequently, the patient performed a 3-min no-load warm-up exercise at 55–65 r/min and then entered an incremental exercise. Based on the patient’s age, height, weight, medical history, and exercise habits, different incremental loads (ranging from 10 to 25 w) were selected until the test was terminated, according to the scientific statement of the American Heart Association. Finally, the patient continued to exercise for approximately 2–3 min during recovery at 30–40 r/min. The system automatically recorded heart rate, blood pressure, and heart and lung data at rest, each exercise stage, and peak exercise and evaluated the degree of conscious exertion at the end of the test (Borg score 6–20 points).

The CPET parameters included peak heart rate (HRmax), peakVO_2_, peak metabolic equivalent (METmax), anaerobic threshold oxygen uptake (AT), PETCO_2_, heart rate reserve (HRR; the difference between the maximum heart rate and rest heart rate), VE/VCO_2_slop, percentage of peakVO_2_ to predicted value (%pred), peak ventilation (VE), peak workload, peak respiratory reserve, peak oxygen pulse, oxygen uptake efficiency slope, and Borg score. The key criterion for selecting the anaerobic threshold is the oxygen uptake at the lowest value of the VO_2_ ventilation equivalent when the VCO_2_ ventilation equivalent is constant, METmax = peakVO_2_/3.50, and other data are directly obtained or calculated by the cardiopulmonary system of COSMED in Italy.

### Collection and follow-up of clinical data

This was a retrospective study. Patients’ medical information were obtained by consulting their medical records and follow-up records, and the participants were determined. Medical information included demographic characteristics, medical examination results, smoking and drinking history, history of hypertension and diabetes, and CPET results. To document whether re-AMI, HF or death occurred after AMI, we conducted follow-ups every 6 months after the patients were discharged from the hospital. If death occurred during this period, follow-up was terminated. Follow-up was conducted via telephone or case review. The follow-up was conducted until August 15, 2023, and the lowest number of follow-up visits among the included patients was 5 and the highest was 14. Causes of death were categorized as obvious cardiac events, unexplained sudden death, or noncardiac causes.

### Statistical analysis

Descriptive statistics were used to describe the baseline data and CPET parameters of the patients, including the mean, standard deviation, number of people, and percentage. The chi-squared test and independent sample *t*-test were used to compare the variables between the no-event and event groups and the re-AMI and HF groups. IBM SPSS software of IBM was used for all analyses. All statistical tests were two-sided, and a *P*-value < 0.05 was regarded as statistically significant.

The data were divided into two sets: 80.0% of the data was used as the training set and 20.0% was used as the test set. Modeling set data from the Department of Cardiovascular Medicine of Zhengzhou Central Hospital Affiliated to Zhengzhou University, Validation set data from the Henan Provincial Hospital of Traditional Chinese Medicine and Anyang District Hospital. According to the results of single factor analysis, the factors with *P* < 0.05 were included in the logistic regression, and the logistic regression equation was established. Furthermore, we used SHapley Additive exPlanations (SHAP) to explain and visualize the effect of predictors based on the risk of adverse outcomes after PCI for AMI.

**Figure 2:**
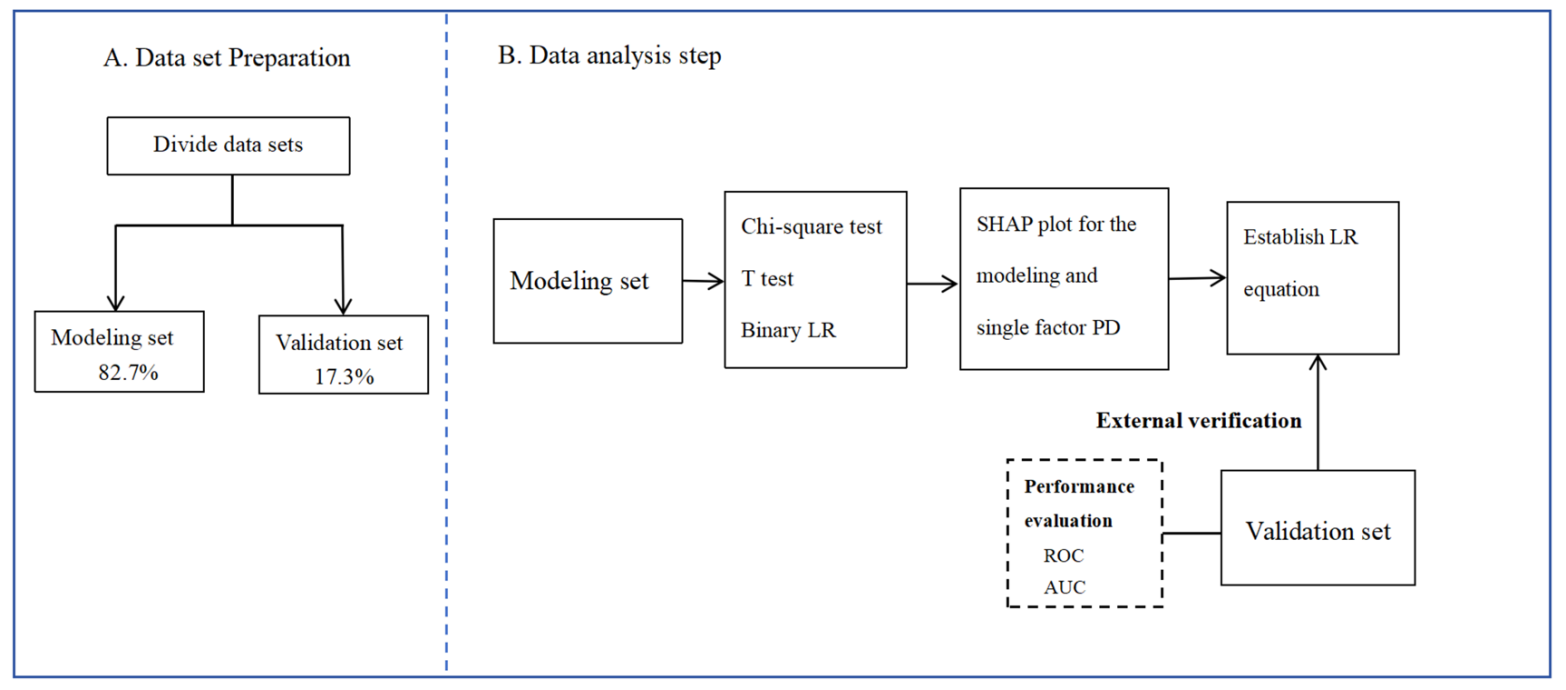
Flowchart summary of our methodology. *LR* logistic regression, *SHAP* SHapley Additive exPlanations, *PD* partial dependence, *ROC* receiver operating characteristic, *AUC* area under the curve. Notes: Modeling set data from the Department of Cardiovascular Medicine of Zhengzhou Central Hospital Affiliated to Zhengzhou University, Validation set data from the Henan Provincial Hospital of Traditional Chinese Medicine and Anyang District Hospital.

The verification data was used for model verification, and the receiver operating characteristic (ROC) curve was drawn for detection. It was considered that the area under the curve (AUC) ˃ 0.50 demonstrated a predictive value, while the AUC ˃ 0.75 demonstrated a relatively high predictive value. The overall flowchart of the analysis is shown in Fig. 2.

## Results

### Comparison of clinical data between the no-event and event groups

The results revealed no significant differences in sex, age, body mass index, left ventricular ejection fraction, clinical diagnosis, blood biochemical data, smoking and drinking history, diabetes history, hypertension history, in-hospital medication (e.g., calcium channel blockers, antiplatelet aggregation drugs, *β*-blockers, and statins), and intervention data between the two groups (*P* > 0.05) (Table 1).

**Table 1:** Comparison of clinical data (x ± s).

Item	No-event group (n = 269)	Event group (n = 41)	*p*
Age (years)	56.53 ± 10.94	59.27 ± 12.79	0.126
Sexuality	M 230 (85.5%) F 39 (14.5%)	M 36 (87.8%) F 5 (12.2%)	0.694
BMI (kg/m^2^)	25.47 ± 3.58	25.95 ± 3.35	0.603
LVEF (%)	60.10 ± 4.94	58.56 ± 5.02	0.531
Clinical diagnosis			
STEMI	135 (50.2%)	28 (68.3%)	0.114
NSTEMI	134 (49.8%)	13 (31.7%)	
Laboratory examination			
Hemoglobin (g/L)	137.75 ± 17.36	137.68 ± 15.35	0.980
BNP (pg/mL)	100.87 ± 135.14	134.06 ± 146.25	0.088
Total cholesterol (mmol/L)	4.12 ± 1.10	3.78 ± 1.20	0.907
Triglyceride (mmol/L)	1.85 ± 2.20	1.74 ± 0.94	0.601
Low-density lipoprotein (mmol/L)	2.40 ± 1.50	2.16 ± 0.90	0.763
High-density lipoprotein (mmol/L)	1.03 ± 0.33	1.04 ± 0.24	0.784
Serum creatinine (µmol/L)	66.79 ± 27.26	65.71 ± 23.06	0.428
Troponin (µg/L)	2.66 ± 6.90	1.09 ± 2.06	0.052
Creatine kinase-MB (U/L)	1.43 ± 2.88	1.25 ± 1.12	0.360
Smoking history			
Never or have quit smoking	159 (59.1%)	21 (51.2%)	0.340
Smoking	110 (40.9%)	20 (48.8%)	
Drinking history			
Never or have quit drinking	188 (69.9%)	25 (60.9%)	0.488
Drinking	81 (30.1%)	16 (39.1%)	
Case history			
Diabetes	60 (22.3%)	10 (24.4%)	0.766
Hypertension	128 (47.6%)	25 (60.0%)	0.131
Medication			
Calcium channel blockers	128 (47.6%)	27 (65.9%)	0.120
Atorvastatin	255 (94.8%)	41 (100.0%)	0.150
β-Blockers	229 (85.1%)	36 (87.8%)	0.688
Coronary artery disease			
Triple vessel disease ≥ 50%	66 (24.5%)	12 (29.3%)	0.515
LAD ≥ 50%	189 (70.3%)	35 (85.4%)	0.176
LCX ≥ 50%	128 (47.6%)	15 (36.6%)	0.188
RCA ≥ 50%	134 (49.8%)	20 (48.8%)	0.902
LMCA ≥ 50%	8 (3.0%)	3 (7.3%)	0.161
Number of brackets	1.26 ± 0.55	1.39 ± 0.59	0.095

Count data in the table represent [number of cases (percentage of total %)]. Compared to the no-event group. *P < 0.05, **P < 0.01. *BMI* body mass index, *LVEF* left ventricular ejection fraction, *STEMI* acute ST-segment elevation myocardial infarction, *NSTEMI* non-acute ST-segment elevation myocardial infarction, *BNP* brain natriuretic peptide, *LAD* left anterior descending branch, *LCX* left circumflex branch, *RCA* right coronary artery, *LMCA* left main coronary artery.

### Comparison of CPET parameters between the no-event and event groups and re-AMI and HF groups

The HRmax, peakVO_2_, METmax, AT, PETCO_2_, HRR, and %pred in the no-event group were significantly higher than those in the event group (*P* < 0.05). The VE/VCO_2_slop in the no-event group was significantly lower than that in the event group (*P* < 0.01). The peakVO_2_, METmax, VE/VCO_2_slop, and %pred were significantly different between the no-event and re-AMI groups (*P* < 0.05). The peakVO_2_, METmax, VE/VCO_2_slop, AT, PETCO_2_, and HRmax were significantly different between the no-event and HF groups (*P* < 0.05). However, there were no statistically significant differences in other parameters, as shown in Table 2.

**Table 2:** Comparison of CPET parameters (x ± s).

Item	No-event group (n = 269)	Event group (n = 41)	Re-AMI group (n = 20)	HF group (n = 25)
HRmax (time/min)	109.51 ± 18.00	102.51 ± 15.29*	102.35 ± 11.96	101.76 ± 16.48*
Peak workload (W)	97.39 ± 30.38	89.12 ± 30.28	93.6 ± 20.80	88.20 ± 30.79
METmax	5.04 ± 1.05	4.22 ± 0.86**	4.42 ± 0.73*	4.13 ± 0.85**
PeakVO_2_ (mL/kg/min)	17.64 ± 3.67	14.73 ± 2.99**	15.37 ± 2.48**	14.44 ± 2.98**
AT (mL/kg/min)	14.73 ± 3.29	12.84 ± 2.54**	13.29 ± 2.49	12.67 ± 2.64**
VE (L/min)	44.28 ± 13.29	41.87 ± 11.95	44.85 ± 10.48	41.64 ± 13.08
Peak oxygen pulse (mL/beat)	11.40 ± 2.86	10.94 ± 2.80	11.90 ± 2.27	10.60 ± 2.86
VE/VCO_2_slop	30.43 ± 4.99	36.10 ± 8.94**	35.78 ± 8.23**	37.83 ± 9.34**
PETCO_2_ (mmHg)	35.77 ± 4.62	33.78 ± 5.39*	34.10 ± 4.23	33.48 ± 5.55**
BR (%)	62.51 ± 9.34	64.07 ± 9.20	64.93 ± 7.48	62.58 ± 10.80
OUES (mL/min/L/min)	1858.71 ± 449.32	1765.73 ± 512.70	1875.50 ± 409.60	1709.32 ± 513.46
Borg	14.86 ± 0.99	15.12 ± 0.93	15.00 ± 1.03	15.04 ± 0.98
HRR (time/min)	38.68 ± 14.76	32.85 ± 13.52*	32.60 ± 10.95	33.36 ± 13.28
%pred	61.71 ± 13.27	55.95 ± 15.53*	53.00 ± 12.81**	58.20 ± 17.84

Compared to the no-event group: *P < 0.05, **P < 0.01.

*HRmax* peak heart rate, *HRrest* rest heart rate, *peakVO*_*2*_ peak oxygen uptake, *METmax* peak metabolic equivalent, *AT* anaerobic threshold oxygen uptake, *PETCO*_*2*_ peak end-tidal carbon dioxide partial pressure, *VE/VCO*_*2*_*slop* carbon dioxide ventilation equivalent slope, *%pred* percentage of peak oxygen uptake to predicted value, *VE* peak ventilation, *BR* peak respiratory reserve, *OUES* oxygen uptake efficiency slope, *HRR* HRmax–HRrest.

MET is based on energy consumption in a quiet environment in the sitting position and it expresses the common index of relative energy metabolism level in various activities, 1MET = oxygen consumption 3.50 mL/(kg·min).

### Survival follow-up

In the adverse event group of 53 patients (14.1%), the first case of re-AMI occurred 122 days after surgery, and the last case occurred 2250 days after surgery. The first case of HF after AMI occurred 62 days after surgery, and the last case occurred 1460 days after surgery. The first death occurred on day 525, and the last on day 2555. Seven patients developed HF after re-AMI.

### Independent factors leading to adverse events after PCI for AMI

According to the results of the above two groups of single factor analysis with or without events, the independent variables with *P* < 0.05 were included in the binary logistic multivariate regression, and the indices included in the binary logistic multivariate regression were selected as the HRmax, peakVO_2_, AT, PETCO_2_, HRR, VE/VCO_2_slop, and %pred (METmax = peakVO_2_/3.50, therefore it was not included in the binary logistic multivariate regression). The forward likelihood ratio method was used to screen variables (including the HRmax, peakVO_2_, AT, PETCO_2_, HRR, VE/VCO_2_slop, and % pred in the equation one by one, and then removing the tests with *P* > 0.05), and the peakVO_2_ (*P* < 0.01, OR: 0.724, 95% CI: 0.568–0.924) and VE/CO_2_slop (*P* < 0.01, OR: 1.169, 95% CI: 1.064–1.284) were identified as independent risk factors for adverse events after PCI for AMI (Table 3).

**Table 3:** Logistic multivariate regression of the three groups.

Group	Item	OR	95% CI		*P*
			Lower limit	Upper limit	
No-event group and event group	PeakVO_2_	0.724	0.568	0.924	0.009
	VE/CO_2_slop	1.169	1.064	1.284	0.001
	Constant	0.000			0.054
No-event group and re-AMI group	VE/CO_2_slop	1.140	1.047	1.242	0.030
	Constant	0.013			0.073
No-event group and HF group	PeakVO_2_	0.705	0.527	0.944	0.019
	VE/CO_2_slop	1.232	1.091	1.391	0.001
	PETCO_2_	1.210	1.028	1.424	0.022
	Constant	0.000			0.016

*peakVO*_*2*_ peak oxygen uptake, *PETCO*_*2*_ peak end-tidal carbon dioxide partial pressure, *VE/VCO*_*2*_*slop* carbon dioxide ventilation equivalent slope.

Based on the results of the univariate analysis of AMI with or without re-AMI after PCI, the independent variables with *P* < 0.05 were included in the binary logistic multivariate regression analysis. The indicators included in the binary logistic multivariate regression were peakVO_2_, VE/VCO_2_slop, and %pred. VE/CO_2_slop (*P* < 0.05, OR: 1.140, 95% CI: 1.047–1.242) was an independent risk factor for re-AMI after PCI for AMI (Table 3).

Based on the results of the univariate analysis of AMI with or without HF after PCI, the independent variables with *P* < 0.05 were included in the binary logistic multivariate regression, and the indicators included in the binary logistic multivariate regression were selected. The HRmax, peakVO_2_, AT, PETCO_2_, and VE/VCO_2_slop variables were selected for binary logistic multivariate regression. The variables were screened by the forward likelihood ratio method: the peakVO_2_ (*P* < 0.05, OR: 0.705, 95% CI: 0.527–0.944), PETCO_2_ (*P* < 0.05, OR: 1.210, 95% CI: 1.028–1.424), and VE/VCO_2_slop (*P* < 0.01, OR: 1.232, 95% CI: 1.091–1.391) were independent risk factors for HF after PCI in patients with AMI (Table 3).

### Analysis of the importance of model factors

According to the American Medical Association cardiopulmonary function diagnostic criteria, the peakVO_2_ was divided into four levels: ≥ 25, ≥ 20–< 25, ≥ 15–< 20, and < 15 mL/min/kg^11^. The VE/CO_2_slop was divided into four levels, comprising < 30, 30–35.90, 36–44.90, and ≥ 45, according to the scientific statement of the European Association for the Prevention and Rehabilitation of Cardiovascular Disease and American Heart Association in 2012^12^. The PETCO_2_ was divided into four levels, including ≥ 37, 30–36, 20–29, and < 20 mmHg. The SHAP model factor importance analysis and single-factor partial dependence were performed on the grouped database.

**Figure 3:**
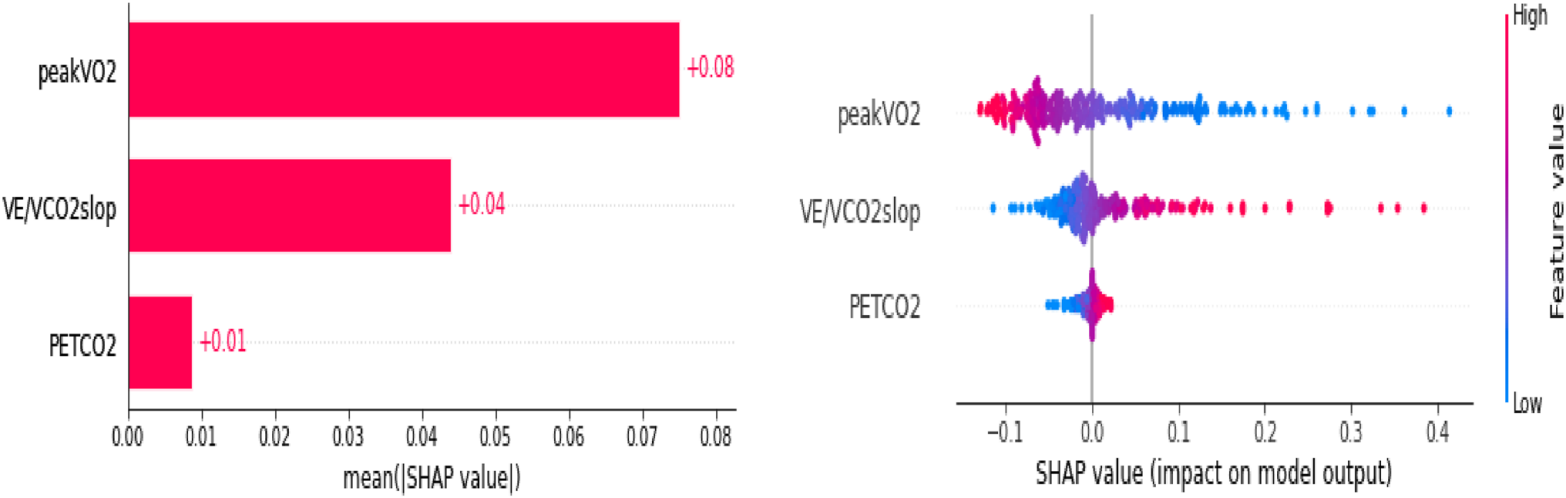
Feature importance in the model. The SHAP value reflects the impact of features in each sample and their positive or negative effects. Contributing factors are ranked in descending order of importance in these plots. Each dot presents a sample; red dots present a higher feature value, and the right side of the vertical line (i.e., feature-specific SHAP values of > 0) presents a higher chance of poor prognosis.

**Figure 4:**
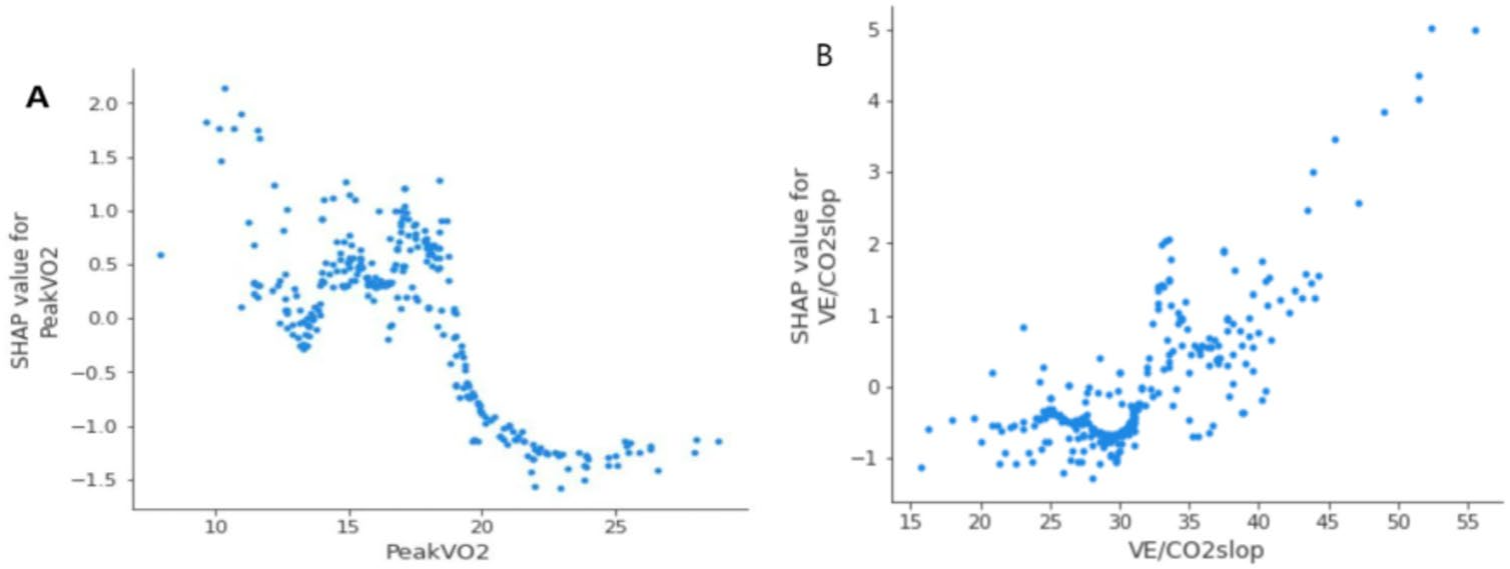
Partial dependence plot of peakVO_2_ and VE/CO_2_slop. The ordinate of the partially dependent graph of SHAP is the SHAP value, and the abscissa is the eigenvalue. The higher the SHAP value, the higher the risk of developing a poor prognosis.

SHAP value analysis showed that peakVO_2_ was the most important factor in the entire model, where the higher the peakVO_2_ value, the lower the probability of an event (Fig. 3). When peakVO_2_ ≥ 20 mL/min/kg (Fig. 4A), the incidence of adverse events was significantly reduced, and the patient prognosis was better. The second most important was the VE/CO_2_slop (Fig. 3), where the higher the value of VE/CO_2_slop, the higher the probability of the occurrence of events. When the VE/CO_2_slop was > 33 (Fig. 4B), the incidence of adverse events increased significantly.

### Establishment of the logistic model

The regression coefficients of peakVO_2_, VE/VCO_2_slop, and PETCO_2_ were established according to the results of the logistic multivariate regression analysis of the three groups. r is the probability of predicting the occurrence of adverse events, and its value is (0–1). The greater the r value, the greater the possibility of adverse events. The regression equation was as follows:

1. With or without event groups: Logit(r) = − 2.415 − 0.170 peakVO_2_ + 0.100 VE/VCO_2_slop.
2. With or without re-AMI groups: Logit (r) =  − 7.765 + 0.157 VE/VCO_2_slop.
3. With or without HF groups: Logit (r) = − 9.942 − 0.231peakVO_2_ + 0.182 VE/VCO_2_slop + 0.148 PETCO_2_.

### Evaluation of the prediction models

**Figure 5:**
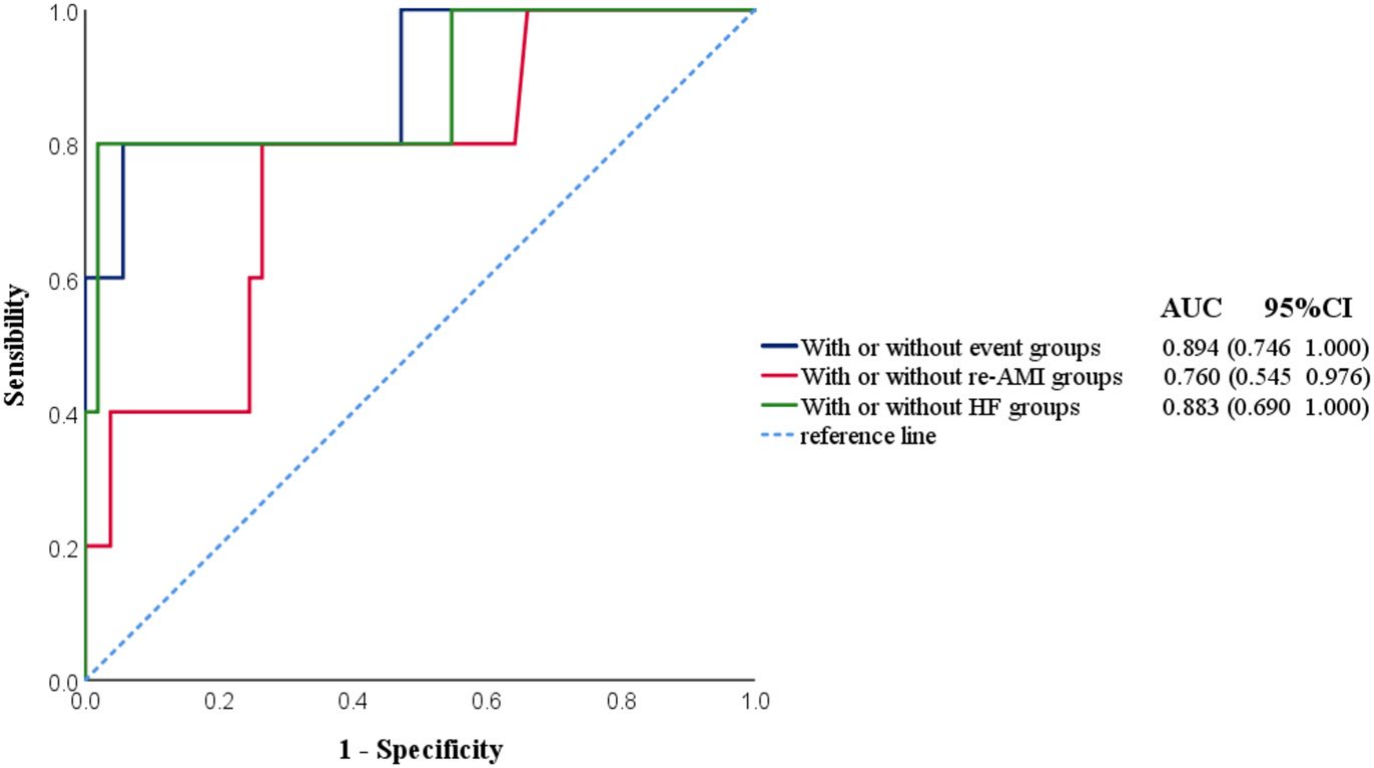
Prognostic index ROC curve of the three groups of patients with AMI after PCI. *re-AMI* re-acute myocardial infarction, *HF* heart failure, *AUC* area under curve, *CI* confidence interval.

The ROC curve was used to evaluate the discriminant ability of the three prediction models. The occurrence of adverse events, re-infarction, and HF after myocardial infarction were used as state variables. According to the established model, the prognostic indices of patients with coronary heart disease after PCI for AMI in this study (data source: the Henan Provincial Hospital of Traditional Chinese Medicine and Anyang Regional Hospital Chest Pain Center) were calculated, and ROC curves were drawn (Fig. 5).

**Figure 6:**
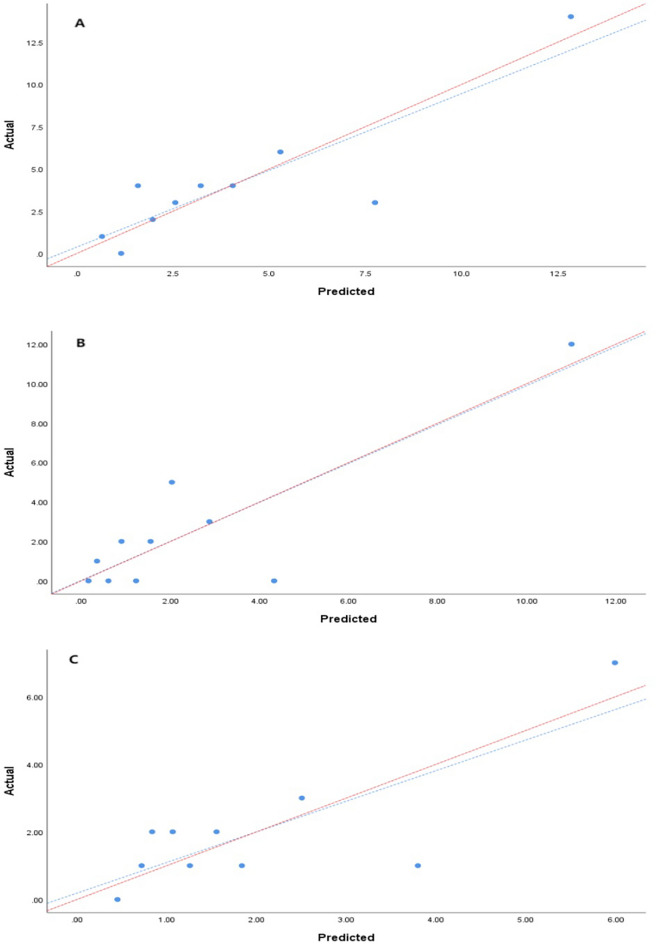
Calibration curve for predicting probability of three prediction models. (**A**) With or without event groups, (**B**) With or without re-AMI groups, (**C**) With or without HF groups.

Prediction model 1: The area under the ROC of the no-event and event groups was 0.894, 95% CI: 0.746–1.000; Prediction model 2: The area under the ROC of the no-event and re-AMI groups was 0.760, 95% CI: 0.545–0.976; Prediction model 3: The area under the ROC of the no-event and HF groups after AMI was 0.883, 95% CI: 0.690–1.000. The three models achieved good performance with AUC > 0.75. The fitness of the prediction model was evaluated by the Hosmer–Lemeshow goodness-of-fit test. The results showed that the *P* values of the three models were 0.255, 0.974, and 0.064 > 0.05, respectively, and the calibration curve was close to the ideal diagonal line (Fig. 6). The prediction models had a good fit and good working effect.

## Discussion

A risk prediction model is a statistical model based on a series of characteristics used to estimate the probability of individual risks or clinical outcomes. In clinical practice, a risk prediction model is primarily used to stratify disease severity and predict disease risk or prognosis. Compared to traditional imaging examinations and noninvasive stress imaging, CPET has great advantages in terms of economy and objective accuracy^13^. More importantly, CPET can be used to obtain a series of parameters closely related to cardiopulmonary function and prognosis^14^. Anuradha Lala et al.^3^ reported that many CPET parameters have a clear predictive value for death in patients with heart disease and rehospitalization in patients with HF.

In this study, we found that the CPET parameters of peakVO_2_, PETCO_2_, and VE/VCO_2_slop were important factors in predicting the prognosis of patients with AMI after PCI. SHAP was used to analyze the importance of the modeling factors, which confirmed that the peakVO_2_, PETCO_2_, and VE/VCO_2_slop had clear predictive values for the recurrence of AMI, HF, hospitalization, and death of patients after PCI. Moreover, peakVO_2_ ≥ 20 mL/min/kg and VE/VCO_2_slop < 33 significantly reduced the risk of adverse prognosis. The ROC curves of the three logistic models were drawn using the verification set, and the results showed that they had good differentiation, a high fit, and a good working effect. The results of internal and external verification showed that the model has good stability and clinical practicability and can be used to predict the prognosis of patients with AMI after PCI.

In 2016, the American medical community officially listed cardiorespiratory fitness (CRF) as a “clinical vital sign.” PeakVO_2_, as an important indicator for evaluating CRF, has become an important parameter for clinical evaluation of patient status, rehabilitation treatment effect, and prediction of life and health. Matsumura et al.^15^ found that the New York Heart Association (NYHA) classification has a good correlation with peakVO_2_ and AT, indicating that HF symptoms are closely related to the body’s ability to transport oxygen. Based on the peakVO_2_ and AT values, Weber and Janicki^16^ more objectively divided the cardiac function of patients with HF into four levels. Most scholars believe that peakVO_2_ and AT are independent predictors of survival in HF, and are more reliable than the NYHA classification or left ventricular ejection fraction^17–19^. Our results also showed that peakVO_2_ was an independent factor affecting the prognosis of AMI after PCI, and a peakVO_2_ ≥ 20 mL/min/kg significantly reduced the incidence of adverse events and improved the prognosis of patients. Therefore, patients with AMI after PCI should initiate aerobic exercises as soon as possible to improve their functional abilities. Functional capacity can be used as a risk factor for predicting death. For every 1 MET increase in functional capacity (3.50 mL/kg/minVO_2_), the risk of death can be reduced by 13.0–35.0%^20^. However, improvements in functional ability are mainly achieved by increasing the peakVO_2_ value through aerobic exercise.

The VE/VCO_2_slop is an indicator of the gas exchange efficiency. Ferreira et al.^21^ found that VE/VCO_2_slop ≥ 43 is an ideal cut-off value for judging the presence of HF; indeed, compared to the classical peakVO_2_-based criteria, it can accurately reclassify 18.3% of HF. Patients with HF with VE/VCO_2_slop ≥ 45 and peakVO_2_ < 10 mL/min/kg have a very poor 4-year prognosis^22^. Studies have shown that aerobic exercise capacity and ventilation efficiency are important reference indicators in evaluating the prognosis of patients with mild obstructive hypertrophic cardiomyopathy^23^. This evidence suggests that the VE/VCO_2_slop can predict the prognosis of patients with cardiovascular diseases. This study also found that the VE/VCO_2_ slope was an independent factor affecting the prognosis of patients with AMI after PCI, where the higher the value, the higher the risk of adverse events. Several studies have confirmed that the ventilation efficiency of patients with coronary heart disease improves after exercise training^24–30^. Additionally, the VE/VCO_2_slop was reduced by 6.0–23.0% in patients with chronic HF after the exercise training program. In this regard, Gademan et al.^27^ showed improvements in the ventilation efficiency of patients with HF after exercise (VE/VCO_2_slop, before training = 35.80 ± 3.90 vs. after training = 31.00 ± 6.10, decreased by 14.0%). In summary, patients with AMI after PCI should start cardiac rehabilitation exercises as soon as possible after achieving a stable condition to increase peakVO_2_ and improve ventilation efficiency.

PETCO_2_ is the end-tidal carbon dioxide partial pressure that reflects pulmonary ventilation and pulmonary blood flow. Arena et al. showed that ventilation efficiency (especially VE/VCO_2_slop) and PETCO_2_ peak during exercise are related to pulmonary hypertension caused by late diastolic dysfunction of left ventricular hypertrophy, and that PETCO_2_ is an important predictor of cardiac-related events in patients with HF^31^. A previous study suggested that PETCO_2_AT is superior to peakVO_2_ in the prognosis of adverse events in patients undergoing CRT^6^. Moreover, Matsumoto et al.^32^ found that in a group of patients with HF, PETCO_2_ at the ventilation threshold was significantly correlated with cardiac output during peak exercise. The sensitivity and specificity of PETCO_2_ (< 38.50 mmHg) in predicting low cardiac output during exercise (cardiac index < 5.11 L/min/m^2^ at peak exercise) were 76.5% and 75.0%, respectively. Tanabe et al.^33^ also reported a significant correlation between PETCO_2_ and cardiac index during peak exercise in patients with HF. The results showed that PETCO_2_ can better reflect the cardiac output response during exercise and has a diagnostic value. Thus, this study found that PETCO_2_ is an independent predictor of HF during follow-up in patients with AMI after PCI, and reported that the risk of HF is 1.21 times higher for every unit increase in PETCO_2_. In summary, PETCO_2_ is not only a prognostic predictor of patients with HF but also a predictor of HF after AMI.

This study has some limitations that warrant discussion. First, this is a retrospective cohort study, and some patients were not included in the analysis because of data loss. Second, during the CPET, some patients did not reach the maximum exercise level owing to the symptom-restricted exercise strategy adopted by individual patients; thus, the HRmax could not be obtained. Lastly, the study participants included patients who underwent CPET in tertiary hospitals, which may not represent the entire AMI population, limiting the generalizability of our results at the grassroots level.

Our results revealed that CPET can well predict the prognosis of adverse events after PCI in patients with AMI and that the risk of adverse events was significantly reduced when peakVO_2_ ≥ 20 mL/min/kg and VE/VCO_2_slop < 33. Therefore, patients with AMI should start aerobic rehabilitation training as soon as possible to promote cardiac function recovery, improve oxygen uptake and ventilation efficiency, reduce the incidence of re-AMI and HF, and improve prognosis.

## Data Availability

The data supporting this comprehensive analysis comes from Department of Cardiovascular Medicine of Zhengzhou Central Hospital Affiliated to Zhengzhou University, Henan Provincial Hospital of Traditional Chinese Medicine, and Anyang District Hospital. Please contact the corresponding author DW.W if needed.
